# Echocardiographic surrogate of left ventricular stroke work in a model of brain stem death donors

**DOI:** 10.1111/eci.14259

**Published:** 2024-06-06

**Authors:** Kei Sato, Louise See Hoe, Jonathan Chan, Nchafatso G. Obonyo, Karin Wildi, Silver Heinsar, Sebastiano M. Colombo, Carmen Ainola, Gabriella Abbate, Noriko Sato, Margaret R. Passmore, Mahe Bouquet, Emily S. Wilson, Kieran Hyslop, Samantha Livingstone, Andrew Haymet, Jae-Seung Jung, Kris Skeggs, Chiara Palmieri, Nicole White, David Platts, Jacky Y. Suen, David C. McGiffin, Gianluigi Li Bassi, John F. Fraser

**Affiliations:** 1 Critical Care Research Group, The Prince Charles Hospital, Brisbane, Queensland, Australia; 2 Prince Charles Hospital Northside Clinical Unit, Faculty of Medicine, University of Queensland, Brisbane, Queensland, Australia; 3 School of Pharmacy and Medical Sciences, Griffith University, Southport, Queensland, Australia; 4 Division of Cardiology, The Prince Charles Hospital, Brisbane, Queensland, Australia; 5 School of Medicine, Griffith University, Gold Coast, Queensland, Australia; 6 Wellcome Trust Centre for Global Health Research, Imperial College London, London, UK; 7 Initiative to Develop African Research Leaders (IDeAL)/KEMRI-Wellcome Trust Research Programme, Kilifi, Kenya; 8 Cardiovascular Research Institute Basel, Basel, Switzerland; 9 Department of Intensive Care, North Estonia Medical Centre, Tallinn, Estonia; 10 Department of Anesthesia, Critical Care and Emergency, Fondazione IRCCS Ca' Granda—Ospedale Maggiore Policlinico, Milan, Italy; 11 Department of Thoracic and Cardiovascular Surgery, College of Medicine, Korea University, Seoul, Republic of Korea; 12 Princess Alexandra Hospital, Brisbane, Queensland, Australia; 13 Faculty of Science, School of Veterinary Science, University of Queensland, Gatton, Queensland, Australia; 14 Faculty of Health, School of Public Health and Social Work, Queensland University of Technology, Brisbane, Queensland, Australia; 15 Cardiothoracic Surgery and Transplantation, The Alfred Hospital, Melbourne, Victoria, Australia; 16 The Department of Surgery, Monash University, Melbourne, Victoria, Australia; 17 Intensive Care Units, St Andrew's War Memorial Hospital, The Wesley Hospital, Uniting Care Hospitals, Brisbane, Queensland, Australia; 18 Medical Faculty, Queensland University of Technology, Brisbane, Queensland, Australia

**Keywords:** brain stem death, heart transplantation, left ventricular stroke work, speckle-tracking echocardiography

## Abstract

**Background:**

The commonest echocardiographic measurement, left ventricular ejection fraction, can not necessarily predict mortality of recipients following heart transplantation potentially due to afterload dependency. Afterload-independent left ventricular stroke work index (LVSWI) is alternatively recommended by the current guideline; however, pulmonary artery catheters are rarely inserted in organ donors in most jurisdictions. We propose a novel non-invasive echocardiographic parameter, Pressure-Strain Product (PSP), as a potential surrogate of catheter-based LVSWI. This study aimed to investigate if PSP could correlate with catheter-based LVSWI in an ovine model of brain stem death (BSD) donors. The association between PSP and myocardial mitochondrial function in the post-transplant hearts was also evaluated.

**Methods:**

Thirty-one female sheep (weight 47 ± 5 kg) were divided into two groups; BSD (*n* = 15), and sham neurologic injury (*n* = 16). Echocardiographic parameters including global circumferential strain (GCS) and global radial strain (GRS) and pulmonary artery catheter-based LVSWI were simultaneously measured at 8-timepoints during 24-h observation. PSP was calculated as a product of GCS or GRS, and mean arterial pressure for PSP_circ_ or PSP_rad_, respectively. Myocardial mitochondrial function was evaluated following 6-h observation after heart transplantation.

**Results:**

In BSD donor hearts, PSP_circ_ (*n* = 96, rho = .547, *p* < .001) showed the best correlation with LVSWI among other echocardiographic parameters. PSP_circ_ returned AUC of .825 to distinguish higher values of cardiomyocyte mitochondrial function (cut-off point; mean value of complex 1,2 O_2_ Flux) in post-transplant hearts, which was greater than other echocardiographic parameters.

**Conclusions:**

PSP_circ_ could be used as a surrogate of catheter-based LVSWI reflecting mitochondrial function.

## 1 Introduction

Heart transplantation (HTx) is a therapeutic option for the management of a selected group of patients with severe cardiac disease.^1–3^ Accurate pre-procurement assessment of donor heart function is essential to predict post-transplant cardiac function and early survival.

The current guideline by the International Society for Heart and Lung Transplantation^4^ mainly focuses on the left ventricular assessment in pre-procurement donor hearts. Echocardiography-based left ventricular ejection fraction (LVEF) is the gold standard for LV assessment in this guideline but its value can be adversely influenced by the LV afterload. The consideration of LV afterload in cardiac assessment is crucially important for the brain stem death (BSD) donors because LV afterload in this cohort drastically increases due to catecholamine storm and subsequently declines due to reduced catecholamine.^5–7^ Therefore, LVEF varies according to the LV afterload, even in the situation where intrinsic cardiac function has minimal change^8,9^ and does not necessarily predict clinical outcomes of recipients following HTx. Therefore, an alternative to LVEF for the assessment of LV function in donor hearts is warranted.^7,10–12^

Left ventricular stroke work index (LVSWI) is a cardiac parameter that integrates cardiac contractility, measured by stroke volume index, with LV afterload, measured by mean arterial pressure. LVSWI is known as an afterload-independent parameter at a given LV end-diastolic volume^13^ and is reported to be a powerful tool to comprehensively assess cardiac performance and predict patients' mortality in the cardiac intensive care.^14^ Indeed, LVSWI is recommended (class IIa) for the donor heart assessment in the current guideline by the International Society for Heart and Lung Transplantation.^4^ However, the accurate measurement of LVSWI requires an invasive pulmonary artery catheter and is rarely used in donor heart assessment due to its invasiveness and various complications such as bleeding and infection.^15^ A non-invasive cardiac measurement as a surrogate for invasively measured LVSWI may improve the assessment of the donor heart function. We proposed a novel speckle-tracking echocardiographic measurement called Pressure-Strain Product (PSP), accounting for both LV contractility and LV afterload. PSP_circ_ and PSP_rad_ are calculated as a product of mean arterial pressure (MAP) and global circumferential strain (GCS) or global radial strain (GRS), respectively. We reported that PSP_circ_ and PSP_rad_ can be surrogate markers of LVSWI in the ovine model of septic cardiomyopathy,^16^ and thus the same concept may be applicable in BSD donor hearts, where both heart pump failure and vascular bed failure can coexist.^17,18^ Furthermore, from the point of cardiac energetics, PSP may correlate with myocardial mitochondrial function as LVSWI does.^19,20^

We hypothesise that PSP (1) correlates with pulmonary artery catheter-based LVSWI, and (2) is associated with cardiomyocyte mitochondrial function. The aim of this study was to test these hypotheses in an ovine model of BSD pre-transplant donor hearts for hypothesis (1) and post-transplant hearts for hypothesis (2).

## 2 Methods

### 2.1 Study design

This study was conducted as a secondary analysis of the study published by See Hoe and colleagues.^21^ The animal experiments were conducted at the Queensland University of Technology (QUT) Medical Engineering Facility (MERF) in Brisbane. Animal ethics was approved by QUT Office of Research Ethics and Integrity (Approval # 16000001109) and ratified by the University of Queensland Animal Ethics Committee (Approval # QUT/393/17/QUT) per the Australian Code of Practice for the Care and Use of Animals for Scientific Purposes and the Animal care and Protection Act 2001 (QLD).

We analysed (1) the correlation between PSP and the pulmonary artery catheter-based LVSWI in BSD donors before HTx, and (2) the area under the curve values to distinguish a higher group of cardiomyocyte mitochondria (cut-off point; mean value of complex 1,2 O_2_ Flux) using the post-transplant hearts 6 h following orthotopic heart transplantation.

### 2.2 Animal model

The details are described in the Appendix —Data S1 of this manuscript. Briefly, female Merino crossbred sheep (1–3 years, 47 ± 5 kg of body weight) were paired and matched based on body weight and blood compatibility. Matched sheep were allocated to be donors or recipients. Donors were randomly allocated to the sham neurologic injury (control; *n* = 16) or brain stem death (BSD; *n* = 15) group, and BSD donor data was used to investigate the correlation between PSP and LVSWI. The data of sham donors was used to investigate the appropriateness of the BSD model. The selected number of 31 is derived from the cases using only one vendor of an echocardiographic machine (31 out of 42 donor cases), while two different vendors were used in the main study previously reported.^21^ This revised inclusion criterion was applied to minimise the intervendor variability in STE analysis as this study primarily focuses on PSP based on the STE strain parameter.

After completion of instrumentation procedures and confirmation of the donor model (sham or BSD), the animals were observed for 24 h, meanwhile, donor heart assessments including both LVSWI and PSP were conducted at pre-defined 8 time points.

For the assessment of the correlation between PSP and mitochondrial function, 30 post-transplanted hearts were investigated. Following the 24-h observation of donors, hearts were procured and preserved by either cold static storage (conventional method using ice, 2 h) or by hypothermic oxygenated perfusion (2 or 8 h). In parallel, the healthy recipient sheep were prepared, and cardiopulmonary bypass (CPB) was established. Following recipient cardiectomy, the donor's heart was implanted using the bicaval orthotopic heart transplantation technique. The recipient was then weaned from CPB and observed for up to 6 additional hours. Echocardiographic parameters and LVSWI were collected at regular intervals; T_baseline_, T_0_ (at the timing of model creation), T_1_, T_3_, T_6_, T_12_, T_18_, T_24_ for donors, and T_0_ (at the timing of off-CPB), T_1_, T_3_, T_6_ in recipients, where T_x_ means x hours after T_0_. A schematic of the experimental timeline is described in Figure S1.

### 2.3 Haemodynamic monitoring

ECG waveform, heart rate and invasive arterial blood pressure through the femoral artery were continuously monitored throughout the experiments. Central venous pressure, stroke volume, cardiac index (CI), and systemic vascular resistance index (SVRI) were continuously monitored via a pulmonary artery catheter. Vasopressor dependency index (VDI) was computed as previously described; VDI = (dopamine + dobutamine + noradrenaline × 100 + adrenaline × 100/MAP, all doses in μg/kg/min),^22^ to determine the amount of vasoactive support that was required to maintain adequate MAP.

### 2.4 Echocardiography

Epicardial echocardiography was performed using X5-1 transducer with an echo stand-off spacer and an IE-33 ultrasound scanner (Philips, Bothwell, WA, USA). Three beat cardiac cycles in the conventional parasternal short axis (PSAX) view with ECG gating were obtained. Conventional apical views (4-chamber, 2-chamber, and long-axis views) could not be obtained due to the anatomical constraints in the ovine model using the short thoracotomy or sternotomy. Especially, non-foreshortened long-axis images were extremely difficult to obtain and thus circumferential and radial strains rather than longitudinal strains were applied for the strain assessment. For the same reason, LVEF was calculated using Teicholz method rather than Simpson's method.

All images were transferred to a separate workstation and analysed offline using TomTec-Arena (TomTec imaging Systems GMBH, Unterschleim, Germany). Feature-tracking analysis for the automated contouring was applied to obtain strain values on appropriate echo loops in the PSAX view, and manual contouring was additionally applied if required. Tracking was then visually assessed for accuracy and the end-diastolic/systolic markers were manually adjusted if required.

Data collected included LVEF, LV global circumferential strain (GCS, %) and global radial strain (GRS, %). In strain analysis using LV short-axis views, considering clinical applicability, only the LV mid-papillary level of the layer rather than entire layers including the base and apex was applied to make the measurement as simple as possible. LV GCS and GRS were defined as the ratio of delta length of myocardial fibre between the end-diastolic and end-systolic phases to the end-diastolic fibre length of circumferential and radial thickness (as strain percentage), respectively.

### 2.5 PSP measurement

**Figure 1:**
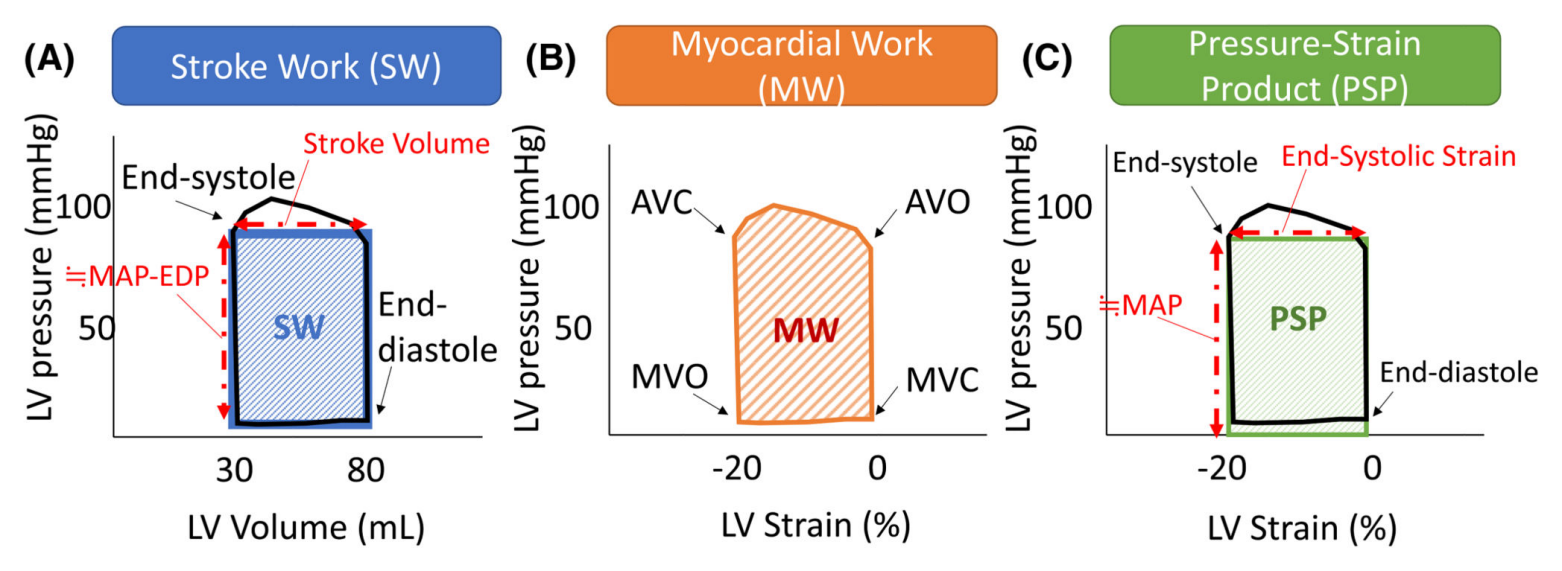
Schematic images of LV stroke work (A), myocardial work (B) and pressure-strain product (C). The area surrounded by blue (A), orange (B) and green (C) diagonals shows estimated stroke work, myocardial work and pressure-strain product, respectively. AVC, aortic valve closure; AVO, aortic valve opening; EDP, end-diastolic pressure; LV, left ventricular; MAP, mean arterial pressure; MVC, mitral valve closure; MVO, mitral valve opening; MW, myocardial work; PSP, pressure-strain product; SW: stroke work.

**Figure 2:**
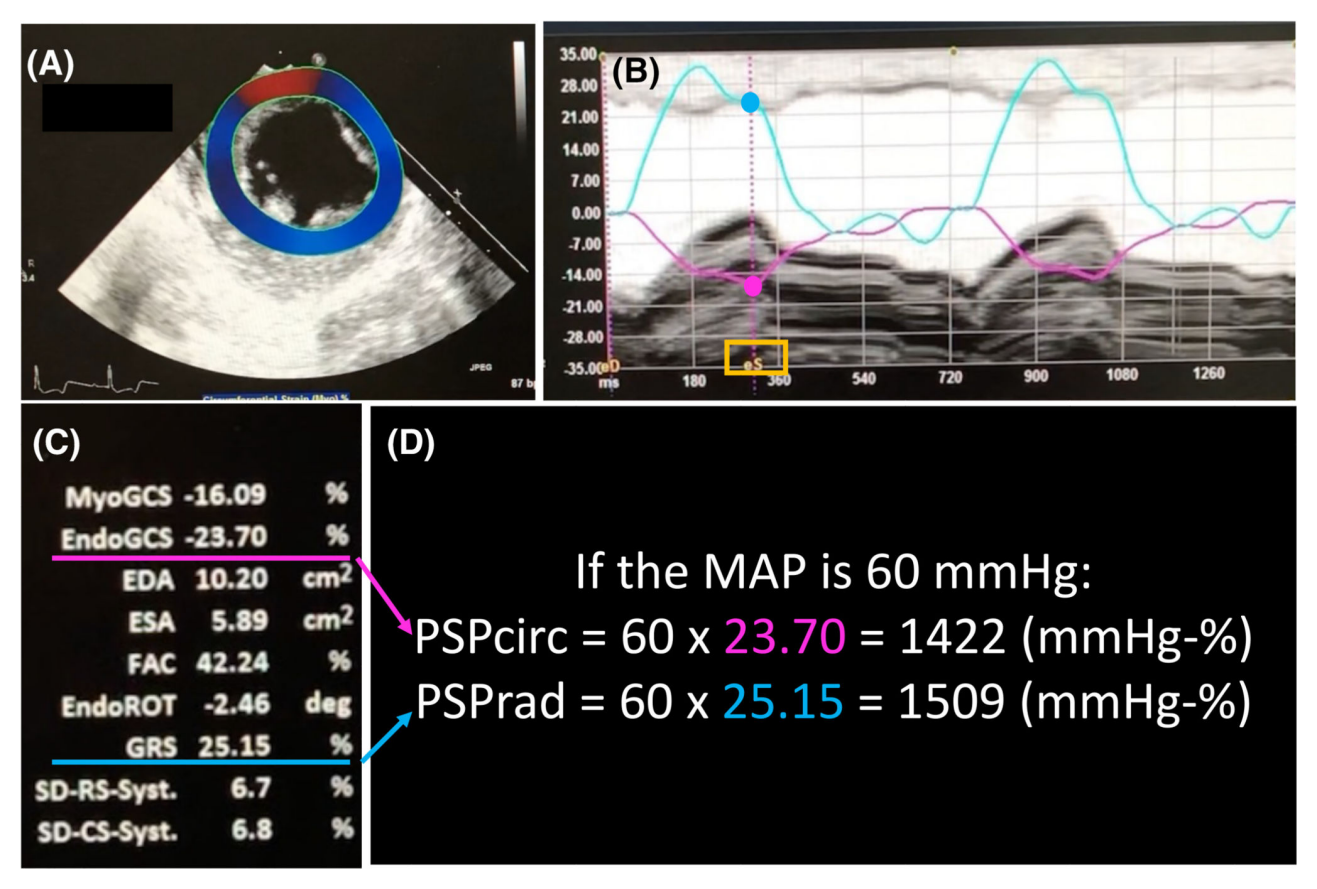
An example of image for the measurement of PSPcirc and PSPrad. (A) Describes LV short axis view at mid-papillary level showing the myocardial layer (dark blue). (B) Describes two strain curves: endo-myocardial GCS (pink) and GRS (light blue). Each value at the end-systolic phase (eS, highlighted in yellow) was used for the calculation of PSPcirc and PSPrad. (C) Describes echocardiographic parameters including myocardial and endocardial GCS, EDA, ESA, FAC, GRS and delta-rotation as the average value from the basal, mid-papillary, and apical levels. (D) Describes the formula for calculating PSPcirc and PSPrad using endocardial GCS and GRS respectively. EDA, end-diastolic area; ESA, end-systolic area; FAC, fractional area change; GCS, global circumferential strain; GRS, global radial strain; MAP, mean arterial pressure; PSPcirc, pressure strain product based on GCS; PSPrad, pressure strain product based on GRS; ROT, rotation; SD-CS, standard deviation of circumferential strain; SD-RS, standard deviation of radial strain.

PSP was calculated by the following formulas: MAP multiplied by GCS (absolute value) for PSP_circ_, or MAP multiplied by GRS for PSP_rad_. The concept of PSP is similar to Myocardial Work, a novel STE parameter described as the area surrounded by an LV pressure-strain loop.^23^ Schematic images of LV stroke work and PSP are illustrated in Figure 1. An example of echocardiographic images and details about PSP calculation is described in Figure 2.

### 2.6 LVSWI measurement

LVSWI is generally calculated by the formula: SVI × (MAP−PCWP) × .0136,^24^ where SVI is stroke volume index and PCWP is pulmonary capillary wedge pressure. We deemed the value of PCWP as zero since PCWP could not necessarily be obtained in all animals due to anatomical peculiarities of sheep pulmonary arterial vasculature.

### 2.7 Mitochondrial function

Mitochondrial function was measured in Oxygraph (Oroboros Instruments, Innsbruck, Austria), which uses high-resolution respirometry to assess the oxygen concentration in the myocardial tissue samples of the left ventricle using post-mortem heart following HTx, and measures mitochondrial oxygen consumption rates.^25^ The details are described in the Supplemental Appendix—Data S1. In short, oxygen consumption over time was measured in cardiac tissue during discrete mitochondrial respiratory states in carbohydrate oxidation; (1) Complex I (C1), (2) Complex II (C2) oxidative phosphorylation (OXPHOS), and (3) LEAK. Oxygen consumption at each state was expressed as O_2_ Flux (pmol/s-mg). Regarding the assessment of the correlation between PSP and mitochondrial function, not the PSP of a single time point but the PSP based on the mean values of four time points (T_0,1,3,6_) in post-transplant hearts were applied because the dose of vasoactive drugs, which might affect PSP, was not the same at each time point.

### 2.8 Statistical analysis

The normality of continuous data was assessed by the Shapiro–Wilk test and parameters were reported as mean ± standard deviation for normally distributed parameters or median and interquartile range (IQR) for not-normally distributed parameters. The normally distributed continuous data was analysed by student-*t* test. No sample size analysis was performed since this was a concomitant observational study.

We investigated the correlation between proposed novel STE parameters (i.e. PSP_circ_ and PSP_rad_) and the catheter-based LVSWI using 15 pre-HTx BSD donor hearts. For this purpose, correlation analysis using Spearman's method was performed as the data of LVSWI was not normally distributed. Linear regression analysis was also added for the assessment of the predictability of PSP for LVSWI. The residual plot and Durbin–Watson statistic were used for the judgement of model appropriateness.

Receiver operating characteristics (ROC) curve analysis was conducted using 30 post-transplanted hearts to investigate the ability of PSP to distinguish a higher or lower group of mitochondrial function (i.e. the O_2_ Flux in Complex 1 + 2), where the mean value of the O_2_ Flux in Complex 1 + 2 was used as a cut-off point. Independent variables include each mean value of PSP_circ_, PSP_rad_, LVEF, GCS and GRS during 6-h observation following HTx (i.e. a mean value of T_0, 1, 3_, and T_6_). The area under the curve (AUC) and *p* values were reported.

Two-way analysis of variance (ANOVA) was used to compare repetitively measured parameters between groups over time. Inter-observer variability for the echocardiographic parameters was analysed by two experienced readers using random cases. Intra-observer variability for the same parameters using the same animals mentioned above was also analysed 3 to 5 years after the initial analysis.

All hypothesis testing is two-tailed and a *p*-value of less than .05 was considered statistically significant. All statistical analyses were performed with SPSS for Mac 29.0 (SPSS Inc, Chicago, USA).

## 3 Results

### 3.1 Studied population

A total of 15 pre-HTx BSD donor hearts were investigated to analyse the correlation between PSP and LVSWI. The data of the other 16 sham donors were included in the supplemental data. Thirty post-transplant hearts were analysed to investigate the relation between PSP and mitochondrial function using the post-mortem samples. The characteristics of haemodynamics and echocardiographic parameters in pre-HTx donor hearts at baseline were described in Table S1.

### 3.2 Correlation between PSP and LVSWI

**Figure 3:**
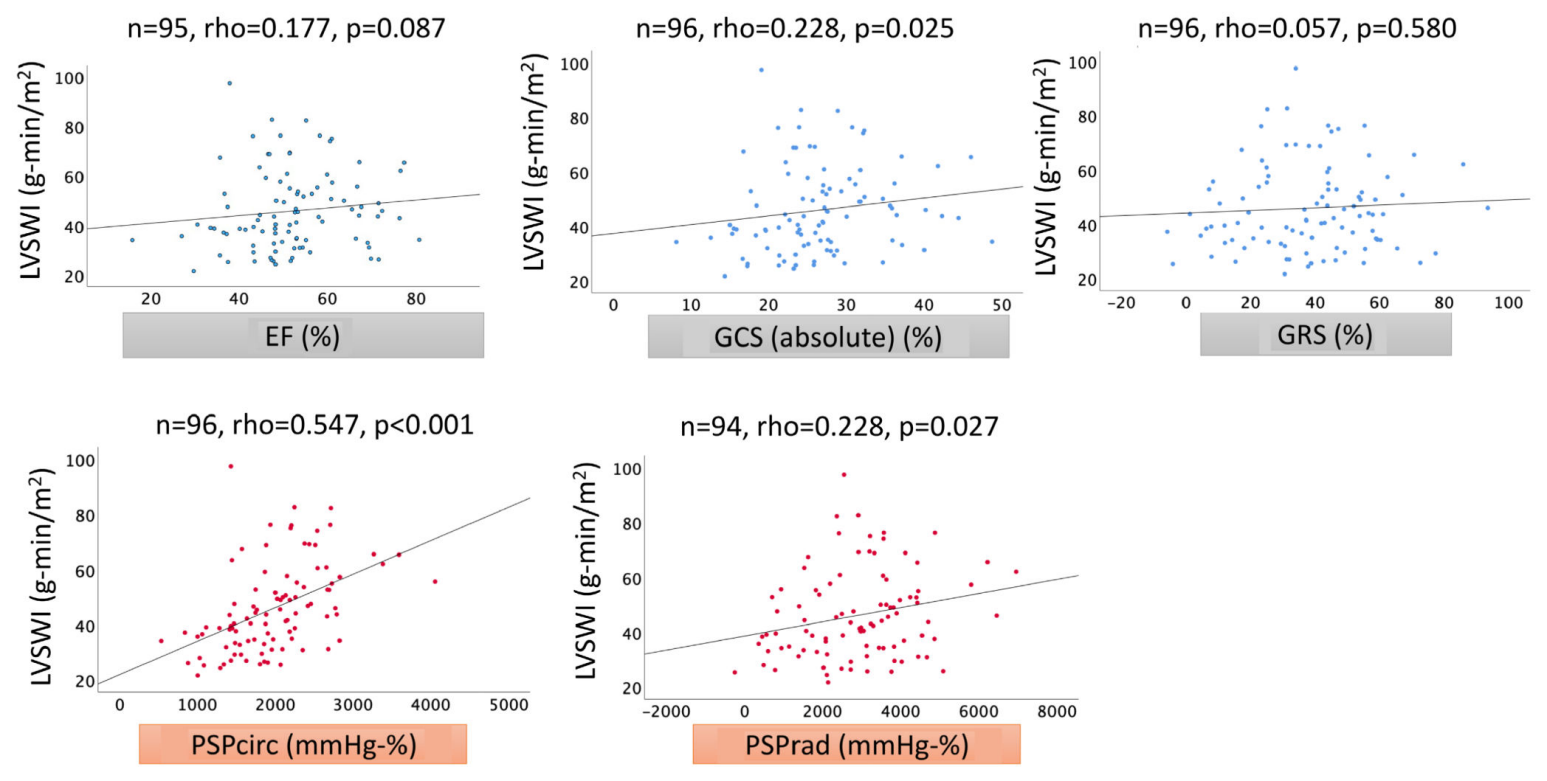
Correlations between catheter-based LVSWI and echocardiographic parameters in BSD donors. Scatter plot graphs between LVSWI and LV EF, GCS, GRS, PSPcirc and PSPrad were described. Each assessment included the valid cases from the time point of baseline to the time point 24 h following the model creation. Correlation analysis was performed by Spearman's method. EF, ejection fraction; GCS, global circumferential strain; GRS, global radial strain; LV, left ventricular; LVSWI, left ventricular stroke work index; PSP (circ), pressure-strain product based on circumferential strain.

In the correlation analysis of BSD donor hearts, PSP_circ_ (rho = .547, *p* < .001) showed the best correlation with LVSWI among other echocardiographic parameters including LVEF (rho = .177, *p* = .087), GCS (rho = .228, *p* = .025), GRS (rho = .057, *p* = .580) and PSP_rad_ (rho = .228, *p* = .027) (Figure 3). In the linear regression analysis, PSP_circ_ (*n* = 96, *B* = .012, confidence interval, CI .007–.017, *p* < .001) showed a statistically significant association with LVSWI, where the Durbin–Watson statistic was 1.728. The residual plot is described in Figure S2.

### 3.3 Myocardial mitochondrial function in post-HTx hearts

**Figure 4:**
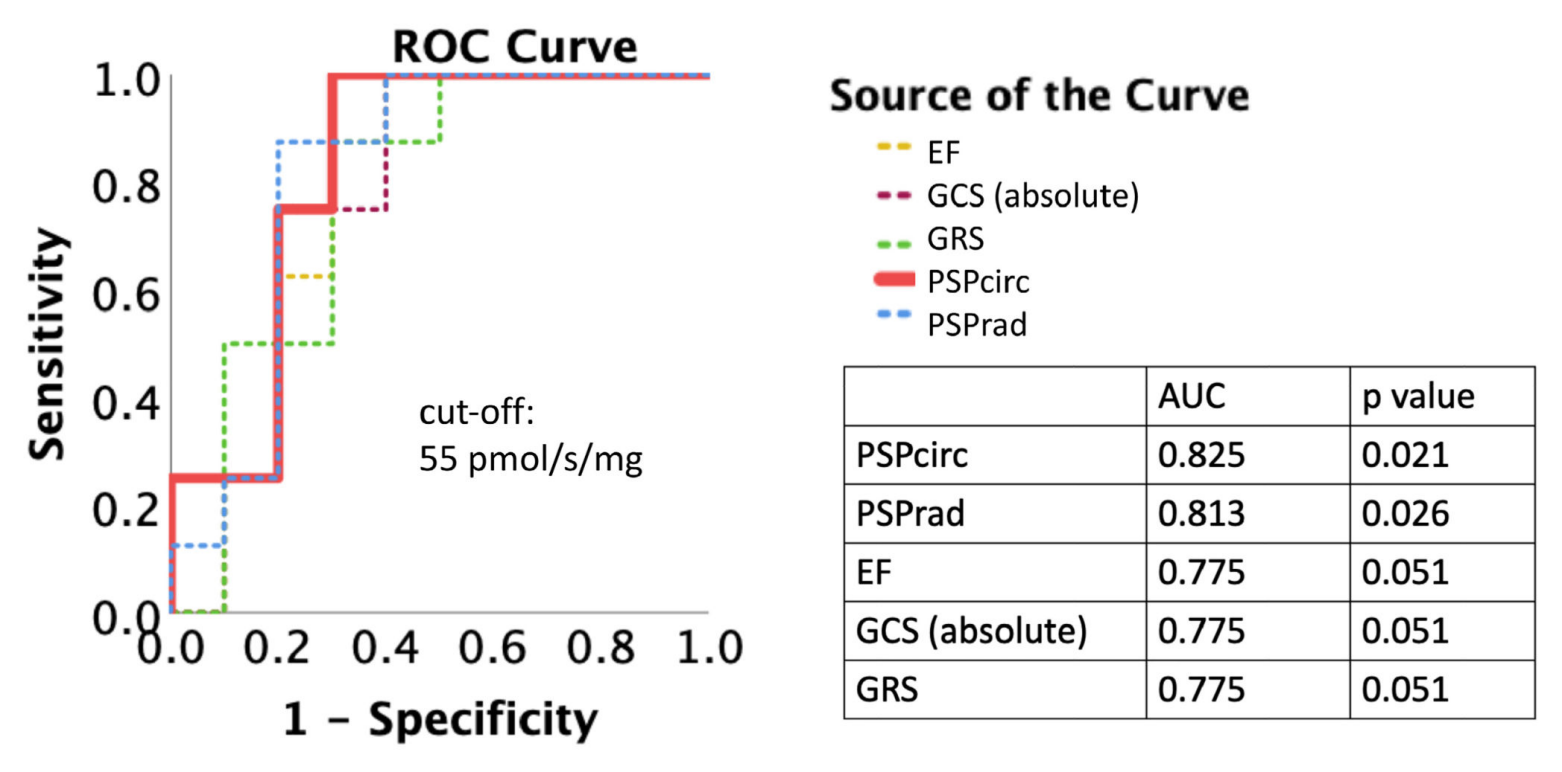
ROC curve analysis to predict mitochondrial function. AUC to predict higher O_2_ Flux (C1 + 2) group (cut-off: mean value of O_2_ Flux C1 + 2; 55 pmol/s/mg). AUC, area under the curve; C1 + 2, Complex1 + 2; EF, ejection fraction; GCS, global circumferential strain; GRS, global radial strain; PSPcirc, pressure-strain product based on circumferential strain; PSPrad, pressure-strain product based on radial strain; ROC, receiver operating characteristics.

The O_2_ Flux in Complex 1 + 2 and LEAK (the value that is not related to ATP generation) in post-transplant hearts are 55 ± 33 pmol/s/mg and 3.7 ± 2.6 pmol/s/mg, respectively. While all echocardiographic parameters (the mean value from T_0_ to T_6_ in post-transplant hearts) showed a correlation with O_2_ Flux (Complex 1 + 2) with statistical significance (Figure S3), PSP_circ_ showed the highest AUC to predict a higher group of O_2_ Flux in C1 + 2 (Figure 4).

### 3.4 Haemodynamics and echocardiographic parameters in donor hearts

MAP and SVRI dropped in the initial 6 h (up to T6) in BSD hearts, and the difference between BSD and sham group was statistically significant despite requiring a higher dose of vasopressors in BSD (Figure S4). BSD donors showed a higher CI than the sham until T6, and then showed the opposite trend until T24 (Figure S4).

Echocardiographic parameters and LVSWI in donors are shown in Figure S5.

### 3.5 Inter- and intra-observer correlation analysis in echocardiographic parameters

Echocardiographic data using 16 cases were randomly chosen for the inter- and intra-observer correlation analysis. Analysis was performed by two experienced readers. There was a statistically significant inter- and intra-observer correlation in PSP_circ_ (Intraclass correlation coefficients, ICC .644, *p* = .003, and ICC .918, *p* < .001, respectively). In PSP_rad_, inter- and intra-observer correlation was less observed than that of PSP_circ_ (ICC 0.460, *p* = .13, and ICC .765, *p* = .002, respectively) (Table S2).

## 4 Discussion

We found that PSP_circ_ correlated with LVSWI in BSD donors (Figure 3), and PSP_circ_ has the potential to predict LVSWI in the linear regression analysis. The linear regression model could be deemed reasonable considering the residual plot (Figure S2) and Durbin–Watson statistic. Compared to the PSP_circ_, PSP_rad_ showed less correlation with LVSWI (Figure 3), which may be due to the wide range of the values based on the variation of GRS (Figure S5).

This study presents a novel finding that the proposed STE strain parameter, PSP, may reflect catheter-based LVSWI and cardiac mitochondrial function. The discovery is of great importance because PSP has the potential to be a more effective measurement than the traditional gold standard LVEF in predicting recipient early mortality. This may support an optimal utilisation of the limited donor hearts.

### 4.1 Clinical significance of PSP

#### 4.1.1 Independence of LV afterload

LVSWI is an afterload-independent parameter at a given preload and thus potentially can reflect intrinsic cardiac function regardless of various variations of LV afterload.^13^ Indeed, LVSWI (>15 g-min/m^2^) is one of the suggested haemodynamic parameters in the current guideline for selecting heart transplant donors.^4^ Stoica et al. reported that, among other catheter-based cardiac parameters (i.e. MAP, central venous pressure, pulmonary capillary wedge pressure and cardiac index), only LVSWI reached a statistically significant difference between the transplanted hearts and rejected hearts, forming the rationale of the current guideline for donor selection.^26^ They suggested that LVSWI is useful until other load-independent indices of ventricular function become clinically available.^26^

The independence of LV afterload is important in the cardiac assessment of BSD donors because this cohort often experiences a significant flux in LV afterload based on the variation of circulating catecholamine.^17^ In this condition, the gold standard parameter, LVEF, cannot always predict the early mortality of recipients following HTx. For example, Chen et al. reported that recipients with reduced LVEF<40% have equivalent 1-year survival compared to recipients with LVEF>50%.^12^ Oras et al. also reported that neither short-term outcomes (30 days mortality) nor long-term (10 years) composite end point of death or re-transplantation over time differed between recipients of donor hearts with versus without LV dysfunction, defined as LVEF<50% and/or regional hypokinesia.^10^ As such, PSP correlating with LVSWI can be independent of LV afterload and represent comprehensive cardiac performance, potentially contributing to a more accurate prediction of recipients' outcomes. Of note, PSP is not entirely equal to LVSWI as seen in Table 1, where LVSWI alone showed a statistical difference between BSD and sham. This difference may derive from the sensitivity to vasoactive drugs such as noradrenaline between stroke volume in LVSWI and strains in PSP. Indeed, in the condition requiring substantial vasoactive support, PSP_circ/rad_ of BSD at T3 was greater than sham, while LVSWI was not. This indicates that we need caution in predicting LVSWI using PSP when requiring a significant amount of vasoactive support (e.g. vasoactive dependency index is around or over 0.5).

#### 4.1.2 Measurement to optimally utilise limited donor hearts

A lack of donor hearts is a critical issue in HTx.^27,28^ The gap between the number of donor hearts and heart transplant candidates continued to grow, reaching more than double in 2015 since 2006.^28^ To efficiently distribute donor hearts to recipients, donor heart function needs to be accurately assessed if it is feasible and durable for the heart transplant. Unfortunately, one of the key metrics to judge the feasibility of donor's hearts, LVEF, does not necessarily predict short- and long-term prognosis post-HTx as mentioned above.

Nevertheless, over two-thirds of presently available donor organs are subjected to discarding,^29^ primarily (around 25% of non-use donor hearts) due to the LV dysfunction assessed by echocardiographic LVEF (independently predicted non-use of donor hearts, after controlling for age, weight and cause of death).^30^ Therefore, there is a need for a more effective measurement than LVEF to optimally utilise the limited donor hearts. PSP as a potential surrogate of LVSWI may express comprehensive cardiac performance regardless of various LV afterload. Clinical studies need to be further investigated on this hypothesis.

### 4.2 A possible rationale and advantage of PSP relating to mitochondrial function

Our study showed that PSP is moderately correlated with mitochondrial function (O_2_ Flux in Complex 1 + 2) (Figure S3), and PSP_circ_ showed the highest AUC among other echocardiographic parameters to predict a higher group of O_2_ Flux in Complex 1 + 2 (Figure 4).

Mitochondria are known as the ‘powerhouse’ of the cell, generating ATP via OXPHOS complexes.^31^ Complex I is thought to play a key role in Ca^2+^ signalling,^31^ which can regulate ATP production by altering the activity of calcium-sensitive mitochondrial matrix enzymes.^32^ Complex II is also a central purveyor of reprogramming metabolic and respiratory adaptation in response to various abnormalities.^33^ As such, O_2_ Flux in Complex 1 + 2 is an important parameter to express the mitochondrial function to produce ATP. Given that LV can use the energy created by mitochondria, it is understandable that PSP as a potential surrogate of LV stroke work is also associated with cardiomyocyte mitochondrial function. This assumption is not against the previous report indicating that mitochondrial dysfunction could be associated with reduced LVSWI.^20^ Lichscheidt et al. also reported that mitochondrial OXPHOS coupling efficiency was related to GLS, one of the STE strain parameters, in the patients post HTx with cardiac allograft vasculopathy.^34^ This result can support our findings where STE strain-based PSP was associated with mitochondrial function. Furthermore, PSP may be able to play an important role in detecting primary graft dysfunction post-HTx, where mitochondrial dysfunction due to the calcium overload through the condition of BSD, the process of HTx operation and following ischemia–reperfusion injuries^35^ may be a factor of LV dysfunction reflecting the autophagy, apoptosis, or necrosis in myocytes.^36,37^

As a caution, we need to recognise that LVSWI can reflect only around 40% of ATP consumption, as other energy is consumed as heat through a basic metabolism.^19,38^ Therefore, LVSWI (or PSP as a surrogate of LVSWI) cannot reflect the whole mitochondrial ATP-generating function.

### 4.3 Limitations

First, this study had a relatively small sample size and thus may not be powered sufficiently to conclude our results. Second, this study may include selection bias because we only included the cases assessed with one vendor (*n* = 31) rather than the mix of two vendors (*n* = 42) of echocardiographic machines to reduce the inter-vendor variability. However, by selecting only one vendor for the echo machine, the disparity of inter-vendor reliability of the echocardiographic data, especially for the STE strains and PSP, was mitigated. Third, we applied GCS and GRS rather than GLS for calculating PSP. Given that GLS is more clinically relevant than GCS and GRS,^39,40^ PSP using GLS may be more suitable for clinical use. Nevertheless, in the case where LV apical views are difficult to obtain like in our study or the access to the designated software to calculate myocardial work is limited, it can be beneficial to have options of PSP_circ/rad_ that correlate with LVSWI. In terms of the strength of the correlation of PSP with LVSWI, whether PSP using strains including entire layers is superior to those using the mid-papillary level alone needs to be further investigated. Last, LVSWI based on pulmonary artery catheter did not account for PCWP, as those values could not be necessarily obtained in all cases due to the anatomical differences of sheep. Therefore, catheter-based LVSWI could have been over-estimated. However, given that the LV function assessed by CI did not significantly decrease with over 3.0 L/min (Figure S4), PCWP might not be extremely high, where the impact of PCWP on LVSWI can be deemed unlikely to be substantial.

## 5 Conclusion

A newly proposed non-invasive PSP correlated with pulmonary artery catheter-based LVSWI, potentially reflecting myocardial mitochondrial function. PSP is expected to improve donor heart assessment and effectively utilise donor hearts, addressing the donor heart shortage. Whether PSP could be a better prognostic predictor of donor hearts than the current gold-standard LVEF needs further investigation.

## Supplementary Material

Supplementary file

## Figures and Tables

**Table 1:** Comparison of cardiac parameters (mean value from T0 to T24) between BSD and sham.

	BSD	*n*	SHAM	*n*	*p* value
EF [%]	51 ± 13	15	50 ± 6	14	.634
GCS [%]	−26 ± 7	15	−25 ± 4	14	.850
GRS [%]	38 ± 15	15	40 ± 8	14	.671
CI [L/min/m^2^]	4.9 ± 0.8	15	4.6 ± 0.7	15	.302
SVI [mL/m^2^]	46 ± 11	15	49 ± 8	16	.434
LVSWI [g-min/m2]	48 ± 12	15	70 ± 17	15	<.001
PSPcirc [mmHg-%]	1865 ± 498	15	2096 ± 432	15	.186
PSPrad [mmHg-%]	2788 ±1047	15	3452 ± 887	15	.071

Abbreviations: CI, cardiac index; EF, ejection fraction; GCS, global circumferential strain; GRS, global radial strain; LVSWI, left ventricular stroke work index; PSPcirc, pressure-strain product based on circumferential strain; PSPrad, pressure-strain product based on radial strain; SVI, stroke volume index.
