# Supplementary material for: Echocardiographic surrogate of left ventricular stroke work in a model of brain stem death donors
Source: Eur J Clin Invest. Author manuscript; Available in PMC 2024 Nov 1. (PMC7616761; doi:10.1111/eci.14259)
Supplement: Supplementary file [file EMS199019-supplement-Supplementary_file.docx]

Supplemental Appendix

**Supplemental Figures**

Supplemental Figure 1. Study timeline.

Supplemental Figure 2. A residual plot in the linear regression analysis between LVSWI and PSP_circ_ in pre-HTx BSD donors.

Supplemental Figure 3. Correlation analysis between O_2_ Flux (C1+2) and echocardiographic parameters.

Supplemental Figure 4. Haemodynamics in donor model (sham vs. BSD).

Supplemental Figure 5. Echocardiographic parameters and LVSWI in donor model (SHAM vs. BSD).

**Supplemental Tables**

Supplemental Table 1. Baseline characteristics.

Supplemental Table 2. Inter- and Intra-observer correlation coefficient in echocardiographic parameters.

**Supplemental Methods**

Supplemental Document 1. The detailed methods in the animal model


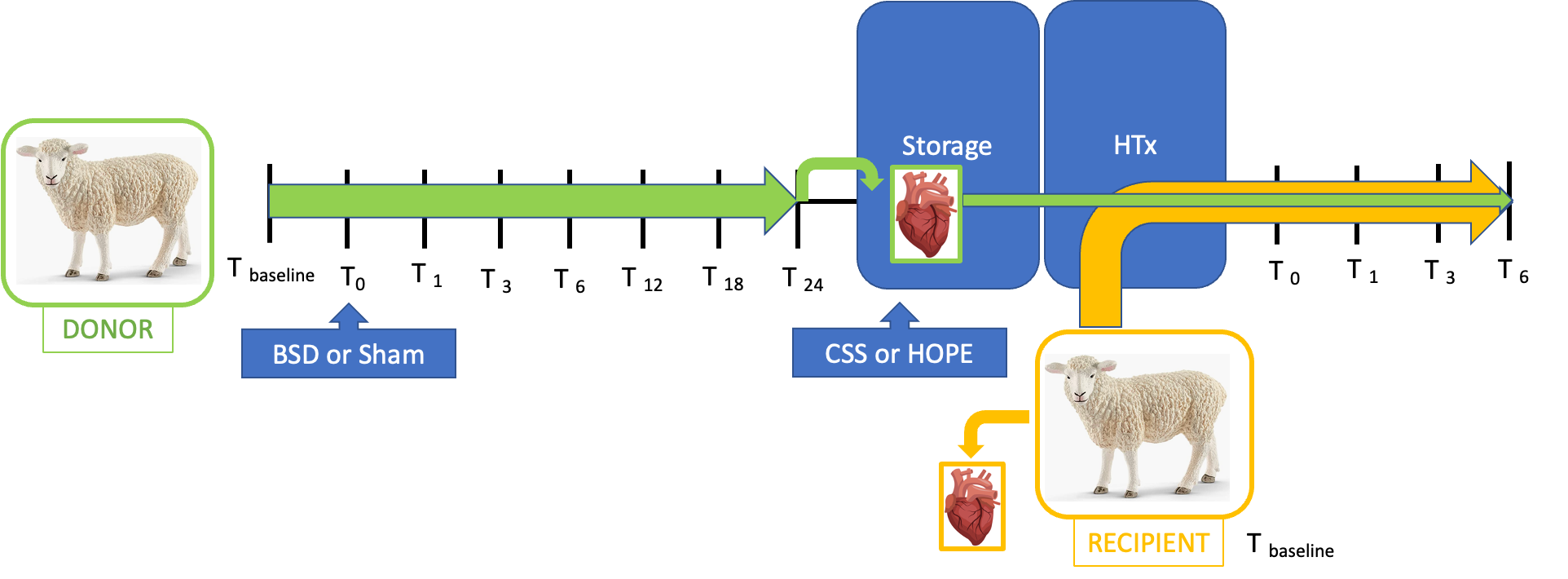


**Supplemental Figure 1. Study timeline.** Following the baseline assessment, a donor model (BSD or sham) was created at T_0_, followed by 24-hour observation. Then, a donor heart was retrieved and preserved in either CSS or HOPE. After removing the original heart of the recipient under cardio-pulmonary bypass, the preserved donor heart was transplanted. Then, a post-transplant heart was observed for up to 6 hours. BSD: brain stem death, CSS: cold static storage, HOPE: hypothermic oxygenated perfusion.

**
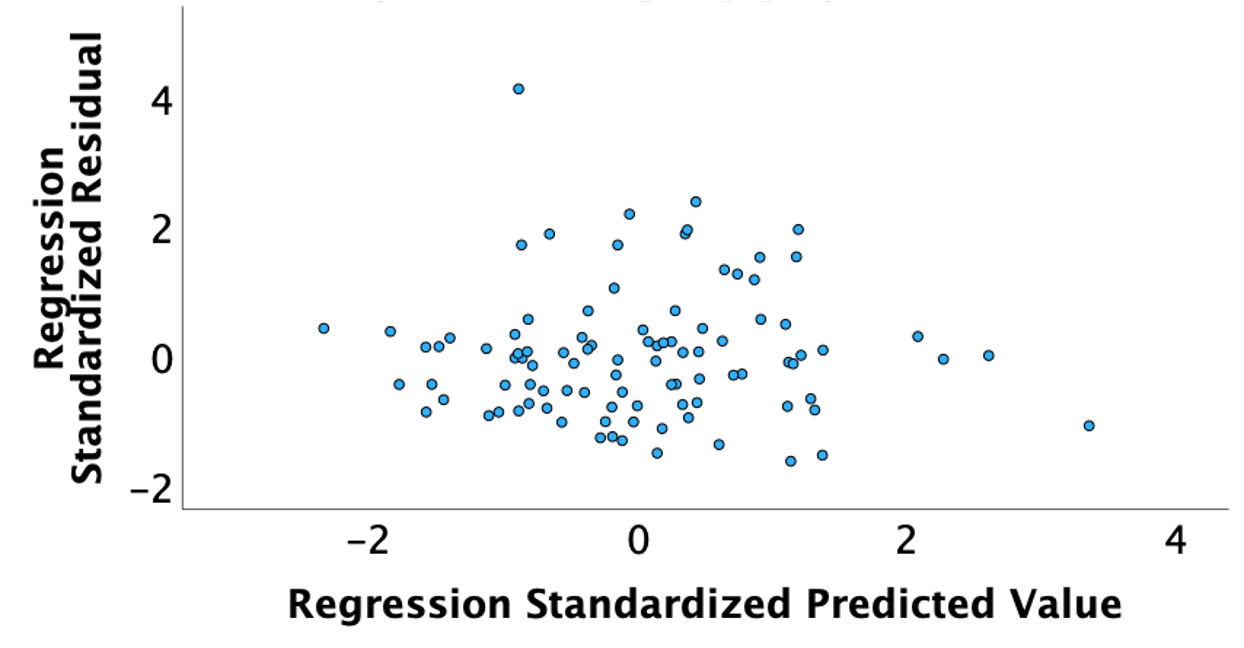
**

**Supplemental Figure 2. A residual plot in the linear regression analysis between LVSWI and PSP_circ_ in pre-HTx BSD donors.** BSD: brain stem death, HTx: heart transplant, LVSWI: left ventricular stroke work index, PSP_circ_: pressure-strain product based on circumferential strain

**
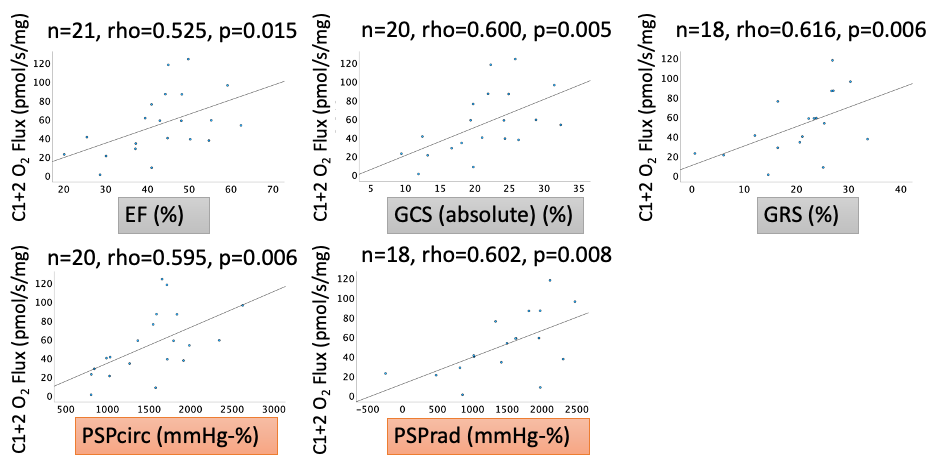
**

**Supplemental Figure 3. Correlation analysis between O_2_ Flux (C1+2) and echocardiographic parameters in post-transplanted hearts.** Scatter plot graphs between O_2_ Flux (C1+2) and echocardiographic parameters (the mean value from T0 to T6 in post-transplanted hearts) are described. Correlation analysis was performed with Spearman's method. T0: the time of successful weaning from cardiopulmonary bypass. T6: 6 hours following T0. C1+2: Complex1+2. EF: ejection fraction, GCS: global circumferential strain, GRS: global radial strain, PSP_circ_: pressure-strain product based on circumferential strain, PSP_rad_: pressure-strain product based on radial strain.


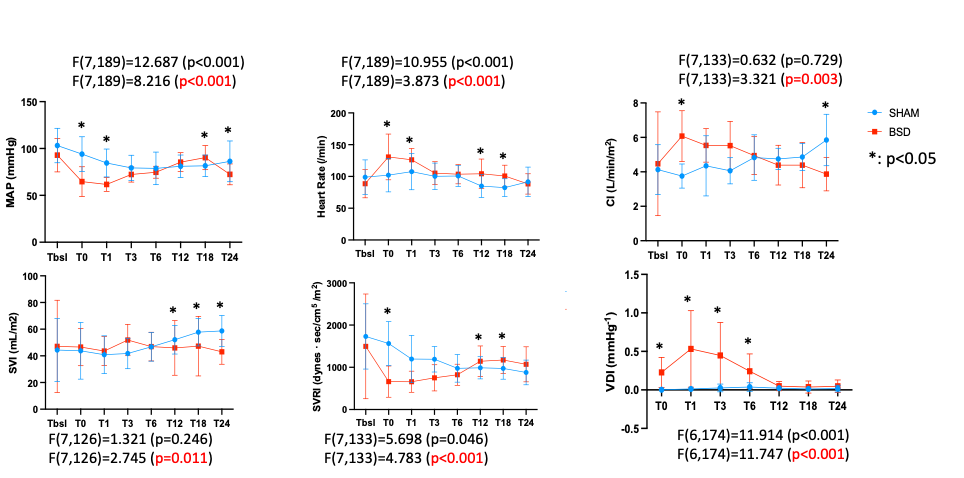


**Supplemental Figure 4. Haemodynamics in donor model (sham vs. BSD).** Haemodynamics in donors (SHAM:n=16, BSD:n=15) is described by MAP, heart rate, CI, SVI, SVRI and VDI. Data are expressed as mean ± SD. Comparison of the effect between groups (lower F and p value) over time (upper F and p value) was performed with general linear models. Tbsl: The time at baseline, T0: the time when the model was created, Tx: x hours after T0. Blue dots = SHAM, red squares = BSD. BSD: brain stem death, CI: cardiac index, MAP: mean arterial pressure, SVI: stroke volume index, SVRI: systemic vascular resistance index, VDI: vasoactive dependency index.

**
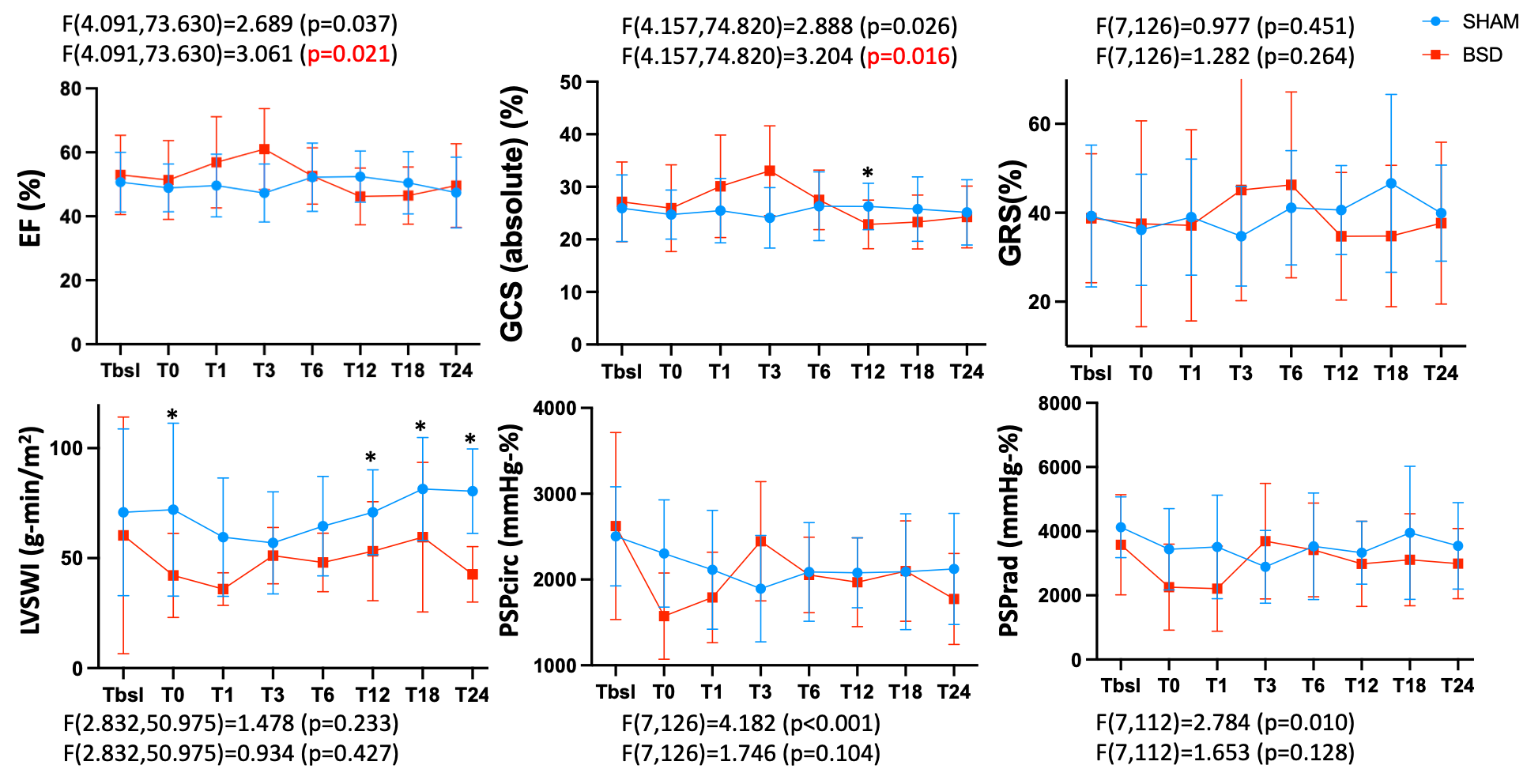
**

**Supplemental Figure 5. Echocardiographic parameters and LVSWI in donor model (SHAM vs. BSD).** LV cardiac parameters in donors (SHAM:n=16, BSD:n=15) are described by EF, GCS, GRS, PSP_circ_, PSP_rad_ and LVSWI. Data are expressed as mean ± SD. Comparison of the effect between groups (lower F and p value) over time (upper F and p value) was performed with general linear models. Tbsl: The time at baseline, T0: the time when the model was created, Tx: x hours after T0. Blue dots = SHAM, red squares = BSD. EF: ejection fraction, GCS: global circumferential strain, GRS: global radial strain, LVSWI: left ventricular stroke work index, PSP_circ_: pressure-strain product based on circumferential strain, PSP_rad_: pressure-strain product based on radial strain.

**Supplemental Tables**

**Supplemental Table 1. Baseline characteristics**

|  | BSD (n=15) | Sham (n=16) |
| --- | --- | --- |
| BW (kg) | 46 (42-52) | 47 (44-49) |
| MAP (mmHg) | 93±18 | 103±18 |
| HR (/min) | 89±22 | 99±27 |
| CI (L/min/m^2^) | 3.5 (3.1-3.8) | 3.9 (3.1-5.0) |
| SVI (mL/m^2^) | 33 (28-64) | 37 (28-56) |
| SVRI (dynes-se/cm^5^/m^2^) | 1307 (584-1788) | 1638 (1381-1982) |
| LVSWI (g-min/m^2^) | 31 (26-82) | 58 (45-95) |
| LV EF (%) | 53±12 | 51±9 |
| GCS (%) | -27±8 | -26±6 |
| GRS (%) | 39±15 | 39±16 |
| PSP_circ_ (%-mmHg) | 2205 (1857-3430) | 2221 (2021-3009) |
| PSP_rad_ (%-mmHg) | 3577±1559 | 41424±950 |

The values are described as mean±SD or median (IQR) as per data distribution. BW: body weight, CI: cardiac index, HR: heart rate, IQR: interquartile range, LV: left ventricular, LVSWI: left ventricular stroke work index, EF: ejection fraction, GCS: global circumferential strain, GRS: global radial strain, MAP: mean arterial pressure, PSP_circ_: pressure-strain product based on circumferential strain, PSP_rad_: pressure-strain product based on radial strain, SD: standard deviation, SVI: stroke volume index, SVRI: systemic vascular resistance index.

**Supplemental Table 2. Inter- and Intra-observer correlation coefficient in echocardiographic parameters.**

| Echocardiographic parameters | Inter-observer ICC | | | Intra-observer ICC | | |
| --- | --- | --- | --- | --- | --- | --- |
|  | Mean | CI | p value | Mean | CI | p vallue |
| LV EF | 0.752 | -0.145 – 0.931 | <0.001 | 0.896 | 0.650 – 0.966 | <0.001 |
| GCS | 0.749 | -0.129 – 0.929 | <0.001 | 0.899 | 0.686 – 0.966 | <0.001 |
| GRS | 0.504 | -0.482 – 0.829 | 0.101 | 0.653 | 0.080 – 0.875 | 0.014 |
| PSP_circ_ | 0.644 | -0.156 – 0.886 | 0.003 | 0.918 | 0.753 – 0.972 | <0.001 |
| PSP_rad_ | 0.460 | -0.657 – 0.816 | 0.134 | 0.765 | 0.333 – 0.918 | 0.002 |

This analysis was conducted using randomly selected 16 animals. CI: confidence interval, EF: ejection fraction, GCS: global circumferential strain, GRS: global radial strain, ICC: intraclass correlation coefficient, LV: left ventricular, PSP_circ_: pressure strain product based on GCS, PSP_rad_: pressure strain product based on GRS.

Supplemental Methodologies

Supplemental document 1. The detailed methods in the animal model

Animal preparation and instrumentation

Animals were fasted at night prior to the experiment with free access to water. Animals were then brought into the operating theatre in a house-made sling. Local anaesthesia (1% Lignocaine) was administered prior to cannulation of the left (8Fr four-lumen central venous catheter) and right (8Fr sheath introducer) extra jugular vein (EJV). Before intubation, Midazolam and Propofol were given as general anaesthesia, and Cefazolin (1g) and Gentamicin (80mg) were intravenously administered for the prophylactic purpose. After intubation with 8-10 mm endotracheal tube, animals were subsequently transferred to the operating table on supine position. ECG, SpO_2_ and end-tidal CO_2_ were continuously monitored, and mechanical ventilation (Hamilton G5, Hamilton Medical, Switzerland) and maintenance anaesthesia (fentanyl: 5 μg/kg/hr, midazolam:0.5-0.8 mg/kg/hr, and ketamine:2.5-7.5 mg/kg/hr) commenced. Respiratory conditions were set and adjusted to achieve the criteria shown in the table 1 below.

| Parameter | Criteria |
| --- | --- |
| Mode | Volume controlled |
| Tidal Volume | 6ml/kg |
| Respiratory rate | ≦35 breaths per minute |
| FiO_2_ | SpO_2_≧90% |
| PEEP | 5-10 cmH_2_O  (adjusted to plateau pressure≦30 cmH_2_O) |
| Plateau pressure | ≦30 cmH_2_O |
| I:E ratio | 1:1 to 1:3 |

**Table 1.** **The setting of mechanical ventilation.** SpO_2_: Blood oxygen saturation, FiO_2_: Fraction of inspired oxygen, PEEP: Positive end-expiratory pressure.

As a fluid infusion, bolus challenge (250-500 ml) was administered depending on the degree of dehydration (judged by serum lactate level and/or echocardiographic assessment), and maintenance infusion of Compound Sodium Lactate (1 mL/kg/h) was started. Nasogastric tube and Yankuer cannula were inserted into the stomach and mouth respectively, to continuously remove excess secretions. Serum electrolytes and glucose levels were controlled according to the target range following the table below (Table 2) as per arterial blood gas analysis.

| Electrolyte | Target range | If abnormalities are detected |
| --- | --- | --- |
| Potassium (K) | 3.5-5.5 mmol/L | Hypokalaemia: Start intravenous infusion of Potassium Chloride (10 mmol/10 ml)  Hyperkalaemia: Stop of supplementation and administration of Calcium gluconate (10 mmol/10 ml) IV |
| Magnesium (Mg) | ≧0.8 mmol/L | Give Magnesium Sulphate 5-10 mmol IV |
| Glucose | ≧1.5 mmol/L | 5 ml bolus of 50% glucose IV |

**Table 2.** **Target range of electrolyte and glucose.**

Two 5Fr arterial cannula were inserted into both femoral arteries for continuous blood pressure monitoring and blood sampling. A pulmonary artery catheter was inserted through the sheath introducer at right EJV. Each haemodynamic parameter was controlled, aiming to achieve the targeted range shown the table below (Table 3).

| Parameter | Target values |
| --- | --- |
| Heart rate (HR) | 50-110 bpm |
| Mean arterial pressure (MAP) | 65-80 mmHg |
| Cardiac index (CI) | ≧2.5 L/min/m^2^ |
| Right atrial pressure (RAP) | 2-10 mmHg |
| Central venous pressure (CVP) | 5-15 mmHg |
| Pulmonary vascular resistance (PVR) | < 250 dynes･sec･ cm^-5^ |
| Mean pulmonary artery pressure (MPAP) | 10-20 mmHg |
| Pulmonary artery wedge pressure (PAWP) | 6-12 mmHg |

**Table 3.** **Target range of hemodynamic parameters**

A 12Fr urinary catheter was inserted to continuously monitor urine output. To maintain a mean arterial pressure (MAP) over 65 mmHg, vasoactive drugs such as noradrenaline (6 mg/100 mL normal saline, 0.025 – 0.2 mcg/kg/min IV), dopamine (1-10 mcg/kg/min), vasopressin (20 units/50 mL 5% Glucose, 0.6 – 2 units/hr IV) or adrenaline (6 mg/100 mL 5% Glucose, 0.1 – 1.5 mcg/kg/min IV) were administered.

Donor-specific procedures and induction of BSD

After administration of vecuronium (0.1 mg/kg iv), a left mini-thoracotomy was performed at the upper border of the 5th rib. A 7Fr three-lumen central venous catheter was inserted into the coronary sinus through the azygos vein, which was used for blood sampling and continuous heparinization.

BSD was induced in donor animals as previously described(1). Briefly, two burr holes on the scalp were created for the purpose of intracranial pressure (ICP) monitoring (4.5mm, left side) and induction of BSD (6.0 mm, right side). Each hole was drilled symmetrically around the intersection of sagittal and lambdoid sutures. Thereafter, a 20G intravenous cannula was inserted into the left 4.5 mm burr hole for ICP monitoring, and a 16Fr Foley catheter (30 mL balloon) was inserted into the right 6.0 mm bur hole. At this stage, 1 hour rest was initiated to stabilize the condition.

In BSD animals, BSD was induced by inflation of the Foley catheter with 10 mL sterile water every 5 min (up to 60 mL). In the event of ST-segment elevation with tachycardia and hypertension, 5 mg of metoprolol was intravenously administered every 2-3 min until ST-segment normalized. BSD was confirmed with 1) high ICP, 2) negative cerebral perfusion pressure (MAP-ICP), 3) loss of haemodynamic response to balloon inflation, 4) loss of pupillary and corneal reflexes, and 5) loss of cough reflex. In Sham donors, a Foley catheter was inserted but not inflated and an additional 30 min rest was added. Time 0 in donors was designated as confirmation of brain death in BSD donors, or completion of 30 min additional monitoring for sham donors.

All donors were monitored for 24 h after T0. Arterial and coronary sinus blood, urine samples and echocardiography were taken at regular intervals. Hormone resuscitation (BSD: methylprednisolone 15 mg/kg, triiodothyronine 4 ug bolus and 3 ug/hr thereafter, vasopressin 1.2 units/hr, Sham: the same doses of methylprednisolone and triiodothyronine, without vasopressin) were administered two hours after T0.

After 24 hours of observation, a median sternotomy was performed, prior to inserting the cardioplegic needle in the ascending aorta. After clamping the ascending aorta, ice-cold St Thomas’s cardioplegic solution (20 mL/kg) was administered through the cardioplegic needle to arrest and preserve the donor heart. The heart was then explanted and preserved either a) on ice in a cooler containing cardioplegia in cold static storage (CSS) group, or b) via hypothermic ex vivo perfusion (HEVP). For HEVP, the donor heart was perfused with oxygenated hyperoncotic cardioplegia (8℃) using a prototype system, in cycles of 15 min on-pump, and 60 min off-pump.

Recipient procedures and cardiopulmonary bypass (CPB)

All recipients were prepared and instrumented in the same manner as donors (see ‘Animal Preparation and Instrumentation’ above). After sternotomy and heparinization (100-300 U/kg, to achieve ACT >400 sec), the aorta was cannulated with a 16Fr elongated one-piece EOPA® cannula, and the superior and inferior vena cava was cannulated with a 24-Fr and 28-Fr right-angled cannula, respectively. Lignocaine (1-2 mg/kg) and amiodarone (5 mg/kg) were administered to prevent arrhythmic complications. Thereafter, cardiopulmonary bypass (CPB) was commenced with a temperature of 32℃. Following ascending aorta cross-clamp, the recipient heart was removed.

Orthotopic heart transplantation (HTx)

Standard orthotopic HTx was performed in recipients using the donor hearts preserved either on ice (CSS) or via HEVP. Anastomoses were performed in the following order: left atrium, inferior vena cava, superior vena cava, pulmonary artery, and ascending aorta. Following the completion of anastomoses and de-airing, the cross-clamp on the ascending aorta was removed. According to the heart condition, defibrillation or a pacemaker was used to support heart rhythm. If the criteria below were met, CPB was weaned; 1) spontaneous or pacing heart rate of 80 to 90 beats/minute, 2) MAP > 60 mmHg without maximal vasoactive support (dopamine > 15 mcg/Kg/min or Epinephrine/Norepinephrine > 0.1 kg/min or vasopressin at any dose), 3) adequate filling of the ventricles as observed through epicardial echocardiography. Residual blood in CPB was transfused, and the recipient was de-heparinised with 20-25 mg of protamine. Following the weaning from CPB, recipient animals were monitored for 6 hours, with blood and urine sampling, and echocardiography at regular intervals. At the end of the study (6 hours additional monitoring post-HTx), animals were euthanized with phenobarbital (0.5 mL/kg, 295 mg/mL IV) and were confirmed as dead by asystole of ECG monitor and null blood pressure. Thereafter, the heart was rapidly retrieved for the purpose of histological analysis(2) and mitochondrial function (heart only). The heart was transported from the surgical room in cold, oxygenated Krebs buffer to the laboratory for further analysis.

Cardiac Tissue Preparation

Under constant carbogen oxygenation of Krebs solution, 3 myocardial biopsies (~10 mg/biopsy) were taken via needle biopsy at the base, mid and apical regions of both ventricles. These biopsies were placed in cold respiration buffer (MiR06) containing (mmol·L-1): 0.5 EGTA, 3 MgCl2·6H2O, 60 K-lactobionate, 2 Taurine, 10 KH2PO4, 20 HEPES, 110 D-sucrose, fatty acid-free bovine serum albumin (1 g·L-1) and catalase (280 U·mL-1) at pH 7.1 [13, 24]. The 3 biopsies, per ventricle, were pooled together to mitigate regional variations in mitochondrial respiration and obtain a more accurate representation of mitochondrial respiration for the entire ventricle. Samples were homogenised using the PBI-Shredder SG3 (Oroboros Instruments) in 500 μL MiR06 (20mg/mL). To yield a 1 mg/mL protein concentration, 50 μL of homogenate was injected into each respiratory chamber of the O2k-oxygraph (Oroboros Instruments, Innsbruck, Austria), followed by respirometry and fluorometry measures.

Mitochondrial function

Mitochondrial function was measured in an Oxygraph (Oroboros Instruments, Innsbruck, Austria), which used high-resolution respirometry (HRR) to assess the oxygen concentration in the myocardial tissue samples and to measure mitochondrial oxygen consumption rates. During carbohydrate oxidation, oxygen consumption was measured during discrete mitochondrial respiratory states i) Complex I (CI) and Complex II (CII) oxidative phosphorylation (OXPHOS), and ii) maximum electron transport system (ETS) capacity. Oxygen consumptions during CI and CII OXPHOS were expressed as O_2_ flux (pmol/s-mg). All measures were corrected for residual oxygen consumption (ROX). Parameters calculated from HRR are briefly described here; 1) CI oxygen flux: Isolated CI phosphorylative state (ATP producing), 2) CII oxygen flux: Isolated CII phosphorylative state, 3) ETS capacity: A measure of maximum mitochondrial oxygen utilization, and 4) LEAK: resting state of non-phosphorylating respiration when oxygen flux is maintained mainly to compensate for the proton leak after inhibition of ATP synthesis(4) (i.e., oxygen flux which is not available for performing biochemical work and thus related to heat production).

References (Only for appendix)

1. Watts RP, Bilska I, Diab S, et al. Novel 24-h ovine model of brain death to study the profile of the endothelin axis during cardiopulmonary injury. Intensive Care Med. Exp. 2015.

2. Xie A, Forrest P, Loforte A. Left ventricular decompression in veno-arterial extracorporeal membrane oxygenation. Ann Cardiothorac Surg 2019;8:9–18.

3. Long Q, Huang L, Huang K, Yang Q. Assessing mitochondrial bioenergetics in isolated mitochondria from mouse heart tissues using oroboros 2k-oxygraph. In: Methods in Molecular Biology.Vol 1966. Methods Mol Biol, 2019:237–246.

4. Gnaiger E. Capacity of oxidative phosphorylation in human skeletal muscle. New perspectives of mitochondrial physiology. Int. J. Biochem. Cell Biol. 2009;41:1837–1845.
